# Are ChatGPT’s Free-Text Responses on Periprosthetic Joint Infections of the Hip and Knee Reliable and Useful?

**DOI:** 10.3390/jcm12206655

**Published:** 2023-10-20

**Authors:** Alexander Draschl, Georg Hauer, Stefan Franz Fischerauer, Angelika Kogler, Lukas Leitner, Dimosthenis Andreou, Andreas Leithner, Patrick Sadoghi

**Affiliations:** 1 Department of Orthopedics and Trauma, Medical University of Graz, Auenbruggerplatz 5, 8036 Graz, Austria; 2 Division of Plastic, Aesthetic and Reconstructive Surgery, Department of Surgery, Medical University of Graz, Auenbruggerplatz 29/4, 8036 Graz, Austria; 3 Department of Dermatology and Venereology, Medical University of Graz, Auenbruggerplatz 8, 8036 Graz, Austria

**Keywords:** artificial intelligence, large language model, periprosthetic joint infection, hip prosthesis, knee prosthesis

## Abstract

Background: This study aimed to evaluate ChatGPT’s performance on questions about periprosthetic joint infections (PJI) of the hip and knee. Methods: Twenty-seven questions from the 2018 International Consensus Meeting on Musculoskeletal Infection were selected for response generation. The free-text responses were evaluated by three orthopedic surgeons using a five-point Likert scale. Inter-rater reliability (IRR) was assessed via Fleiss’ kappa (FK). Results: Overall, near-perfect IRR was found for disagreement on the presence of factual errors (FK: 0.880, 95% CI [0.724, 1.035], *p* < 0.001) and agreement on information completeness (FK: 0.848, 95% CI [0.699, 0.996], *p* < 0.001). Substantial IRR was observed for disagreement on misleading information (FK: 0.743, 95% CI [0.601, 0.886], *p* < 0.001) and agreement on suitability for patients (FK: 0.627, 95% CI [0.478, 0.776], *p* < 0.001). Moderate IRR was observed for agreement on “up-to-dateness” (FK: 0.584, 95% CI [0.434, 0.734], *p* < 0.001) and suitability for orthopedic surgeons (FK: 0.505, 95% CI [0.383, 0.628], *p* < 0.001). Question- and subtopic-specific analysis revealed diverse IRR levels ranging from near-perfect to poor. Conclusions: ChatGPT’s free-text responses to complex orthopedic questions were predominantly reliable and useful for orthopedic surgeons and patients. Given variations in performance by question and subtopic, consulting additional sources and exercising careful interpretation should be emphasized for reliable medical decision-making.

## 1. Introduction

The interactive chatbot ChatGPT (OpenAI. “ChatGPT.” OpenAI’s GPT-3.5 model. 2021. https://openai.com/, accessed on 20 May 2023) is a language-based artificial intelligence (AI) model powered by the advanced GPT-3.5 language model in the free version and has been trained using deep learning techniques on a vast corpus of textual data from online sources current up to September 2021 [1,2]. Recently, it has been raising attention in the medical community due to its impressive contextual understanding and coherent conversational abilities, allowing it to generate human-like responses to various topics [3,4,5,6,7].

ChatGPT has shown promising results in correctly answering medicine-related multiple-choice and single-choice questions [8,9,10,11], including examinations such as the United States Medical Licensing Examination (USMLE) and the German state examination in medicine [10,12,13]. Moreover, ChatGPT has been acknowledged as a reliable and useful tool for providing information on common rheumatic diseases [3]. Although these examples highlight the value of AI-generated medical knowledge in guiding patients and supporting medical professionals, it is important to acknowledge that as the complexity of questions and scenarios escalates, it becomes imperative to thoroughly evaluate the knowledge and accuracy of AI models to ascertain their reliability in medical decision-making and feasibility in widespread adoption [3,6,14,15].

Presently, a comprehensive investigation into ChatGPT’s performance in complex medical assessments, including an evaluation of its generated responses by experts, specifically within the field of arthroplasty, is still lacking [16]. Although previous research suggests that ChatGPT’s testing performance and knowledge are comparable to that of a first-year orthopedic surgery resident [16], addressing this knowledge gap is critical if ChatGPT is to fulfill its potential as a valuable resource for orthopedic surgeons and patients seeking insights on complex orthopedic topics.

Therefore, this study aimed to assess the performance of ChatGPT’s free-text responses when the model was confronted with complex orthopedic questions related to periprosthetic joint infections (PJI) of the hip and knee. The objective was to evaluate whether ChatGPT provides trustworthy information for PJI prevention, diagnosis, treatment, and outcomes.

## 2. Materials and Methods

The study was conducted in adherence to the ethical standards outlined in the Declaration of Helsinki. As the study did not involve human or animal data, ethics committee approval was not required.

For the purpose of this study, data from the 2018 International Consensus Meeting (ICM) on Musculoskeletal Infection, which took place from 25 to 27 July 2018 at Thomas Jefferson University in Philadelphia, Pennsylvania, were utilized [17]. A subset of 27 questions (Q1–27) out of a total of 155 from the Hip & Knee subsection of the 2018 ICM was directed to ChatGPT (OpenAI. “ChatGPT.” OpenAI’s GPT-3.5 model. 2021. https://openai.com/ accessed on 20 May 2023) for the purpose of generating free-text responses on PJI of the hip and knee (Table 1). Among these questions, eight (Q1–8) were related to PJI prevention, and five (Q9–13) focused on PJI diagnosis, while one question each (Q14 and Q15) addressed pathogen factors and fungal PJI, respectively. Furthermore, 11 questions (Q16–26) were directed toward the treatment of PJI, and one question (Q27) pertained to PJI outcomes, resulting in a total of six different subtopics. The specific questions included in this study and their corresponding official recommendations can be accessed at the following link: https://icmphilly.com/hip-knee/ accessed on 20 May 2023. The data retrieval and generation of responses took place on 20 May 2023.

To ensure a systematic approach, each of the included 27 questions was assigned to an individual chat session within the ChatGPT interface. The process of selecting a single question per (sub-)section of the Hip & Knee 2018 ICM adhered to a predefined set of criteria. First, one question was chosen to represent each (sub-)section of the Hip & Knee 2018 ICM. Second, the main questions selected for inclusion and statistical analysis were those with the highest level of agreement among the delegates from 93 countries who participated in an electronic voting process used to decide their agreement on the recommendations made during the 2018 ICM [17]. When multiple questions received equal agreement ratings, preference was given to the question supported by a higher level of evidence related to the recommendations. When levels of consensus and levels of evidence were identical for several questions, the question with the lowest abstention rate was prioritized. To ensure accurate and organized documentation, each response generated by ChatGPT was recorded by copying and pasting it into a dedicated text file. The responses were systematically collected under their respective questions and recommendations. This text file served as a comprehensive record for the study. Subsequently, the file was shared with the independent raters for evaluation. The evaluation was conducted based on the recommendations of the 2018 ICM and the evaluators’ medical and scientific expertise.

The reliability and relevance of each response were evaluated by three independent raters: P.S, G.H., and S.F.F., three board-certified orthopedic surgeons specializing in hip and knee surgery, with 13, 7, and 10 years of expertise, respectively. The evaluation employed a five-point Likert-type scale (Table 2).

To avoid bias, the assessment was conducted in separate settings, guaranteeing that one rater’s judgment did not influence the judgment of another. The evaluation considered various aspects, as has been previously described [18], including the completeness of the provided information (Completeness), the presence of misleading content (Misleading) and factual errors (Errors), the timeliness of the information (Up-to-dateness), and its suitability as a resource for patients (Patients). Moreover, we extended our assessment to include the information’s suitability for orthopedic surgeons (Surgeons) (Table 2).

Data were subjected to statistical analysis using the IBM Statistical Package for the Social Sciences (SPSS, Version 27.0; IBM, Armonk, NY, USA) software. Alongside the calculation of means and standard deviations (SD), Fleiss’ kappa values (FK) and 95% confidence intervals (CI) were employed to assess inter-rater reliability (IRR) among all three raters. To ensure consistent terminology in characterizing the degree of agreement within the context of kappa statistics, Landis and Koch have proposed a benchmark scale for interpretation [19]. According to this scale, a kappa value below 0.00 signifies poor agreement, kappa values ranging from 0.00 to 0.20 indicate slight agreement, kappa values ranging from 0.21 to 0.40 suggest fair agreement, and kappa values ranging from 0.41 to 0.60 reflect moderate agreement. Substantial agreement is denoted by kappa values ranging from 0.61 to 0.80, while an almost perfect agreement is indicated by kappa values ranging from 0.81 to 1.00. Values of *p* less than 0.05 were considered statistically significant.

## 3. Results

### 3.1. Overall Total Agreement

**Figure 1:**
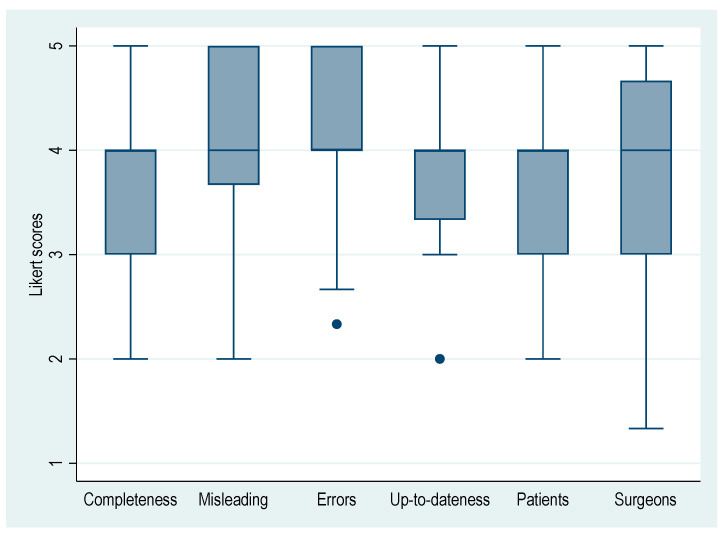
This figure shows a boxplot of the six evaluated aspects across the 27 questions assessed (Q1–27) by all three raters. Likert scale: 1 = strongly agree, 2 = agree, 3 = neutral, 4 = agree, 5 = strongly disagree. The Likert scale is reversed for the aspects “Misleading” and “Errors”. Median and interquartile range [IQR]: Completeness (4.00 [3.00, 4.00]), Misleading (4.00 [3.75, 5.00]), Errors (4.00 [4.00, 5.00]), Up-to-dateness (4.00 [3.75, 4.00]), Patients (4.00 [3.00, 4.00]), Surgeons (4.00 [3.00, 5.00]).

The overall agreement among all three raters demonstrated a substantial level of IRR (FK: 0.706, 95% CI [0.649, 0.763), *p* < 0.001), with a mean (±SD) Likert score of 3.87 ± 0.66, suggesting a tendency towards ChatGPT’s free-text responses to PJI of the hip and knee being generally perceived as complete, not misleading, having occasional factual errors, and suitable for both patients and orthopedic surgeons. An overview of the evaluated aspects across the 27 questions is presented in Figure 1.

### 3.2. Agreement on Evaluated Aspects

The results of the inter-rater reliability and agreement analysis for the combined set of analyzed questions (Q1–27) based on the assessed aspects among all three raters and the two experts are displayed in Table 3. Mean Likert scores, standard deviations (SDs), and FK values were employed to evaluate different aspects associated with the responses generated by ChatGPT.

The assessment of IRR revealed an almost perfect level of agreement among the evaluators regarding the completeness of the information (Completeness) and presence of relevant factual errors. The highest mean (±SD) Likert score (4.14 ± 0.58) and FK value (0.880, 95% CI [0.724, 1.035], *p* < 0.001) were observed for the aspect of factual errors (Errors), indicating that the experts tended to disagree with the proposition that there were relevant factual errors provided by ChatGPT. Regarding completeness of the content, this aspect obtained the fourth-highest mean (±SD) Likert score (3.80 ± 0.63) and the second-highest FK value, 0.848 (95% CI [0.699, 0.996], *p* < 0.001).

The evaluations concerning the presence of misleading information (Misleading) and patient suitability (Patients) indicated a substantial IRR (*p* < 0.001 for both). The mean (±SD) Likert score of 4.04 ± 0.67 suggests that the raters predominantly disagreed with the idea that ChatGPT provides misleading information. Similarly, it was generally agreed that the information provided was suitable for patients, as evidenced by a mean (±SD) Likert score of 3.69 ± 0.64.

When considering the timeliness (Up-to-dateness) of ChatGPT’s responses and their suitability for orthopedic surgeons, the mean (±SD) Likert scores of 3.90 ± 0.45 and 3.63 ± 0.95, respectively, suggest a strong tendency towards agreement, with a moderate level of IRR for both aspects (*p* < 0.001 for both).

### 3.3. Agreement Based on Individual Questions (Q1–27)

Detailed data on the three raters’ evaluations for each question (Q1–27) are listed in Table S1. Means ± SD, FK values, and the 95% CI for each question are presented in Table 4.

Among the individual questions, the potential contamination of the surgical field by particles (Q5) achieved the highest mean Likert score, 5.00 ± 0.00, indicating a strong agreement on the content’s trustworthiness among the three raters. This was further supported by an FK value of 1.000, denoting near-perfect IRR. On the other hand, the question involving the differentiation in treatment and management between early postoperative infection and acute hematogenous infection (Q16) obtained the lowest mean (±SD) Likert score, 1.94 ± 0.42, suggesting low trustworthiness of the provided information. The FK value for Q16 was 0.234 (95% CI [−0.134, 0.602]), indicating a non-significant poor level of agreement (*p* = 0.212).

### 3.4. Inter-Rater Reliability (IRR) Based on Subtopics

The IRR varied across the questions of the six different subtopics (Table 5): (I) (PJI) Prevention, (II) Diagnosis, (III) Pathogen Factors, (IV) Fungal PJI, (V) Treatment, and (VI) Outcomes.

Free-text responses to prevention-related questions exhibited the highest mean (±SD) Likert score (4.53 ± 0.53), with a significant substantial level of IRR (FK: 0.685, 95% CI [0.528, 0.842], *p* < 0.001). Likewise, a significant substantial IRR was observed for responses related to PJI diagnosis and PJI treatment (*p* < 0.001 for both). Although the responses to treatment-related questions showed a substantial IRR, the mean (±SD) Likert score was the lowest for this subtopic overall (3.54 ± 0.95).

Non-significant moderate levels of IRR were observed for responses to fungal PJI (FK: 0.446, 95% CI [−0.016, 0.908], *p* = 0.058) and pathogen factors (FK: 0.438, 95% CI [−0.024, 0.899], *p* = 0.063), with mean (±SD) Likert scores of 4.28 ± 0.46 and 3.89 ± 0.32, respectively. Outcome-related responses yielded the second-lowest mean (±SD) Likert score (3.61 ± 0.68) and lowest IRR (FK: 0.299, 95% CI [−0.095, 0.693], *p* = 0.137), suggesting low trustworthiness.

## 4. Discussion

The study’s objective was to evaluate the performance of ChatGPT, a generative pre-trained transformer (GPT) language model, on providing answers to complex orthopedic questions derived from the Hip & Knee 2018 International Consensus Meeting (ICM) on periprosthetic joint infections (PJIs) of the hip and knee.

Our study showed that there were diverse levels of inter-rater agreement across the evaluated aspects, leading to a partial rejection of the notion that ChatGPT would not provide reliable information for preventing, diagnosing, and treating PJIs of the hip and knee. The presence of factual errors and the completeness of the content supplied were aspects in which we observed the highest level of IRR across the raters, indicating a more consistent evaluation in these areas. The lowest IRR (moderate level IRR) was found concerning the up-to-dateness of the information and its suitability for orthopedic surgeons.

However, in an overall assessment, ChatGPT was generally perceived as complete, not misleading, having minor factual errors, up-to-date, and valuable for patients and orthopedic surgeons. These findings are comparable to the conclusions of a prior investigation conducted by Uz and Umay [3], which assessed the reliability and usefulness of ChatGPT’s free-text answers about keywords related to common rheumatic diseases. The evaluation involved the use of two seven-point Likert-type scales, ranging from “not useful at all” and “completely unsafe” to “extremely useful” and “absolutely reliable”, respectively [3]. According to their findings, ChatGPT can be regarded as a reliable source of information that is useful for patients [3], a finding which aligns with our results, as evident in the overall mean ± SD Likert score of 3.70 ± 0.64 and the substantial level of IRR (FK: 0.627, 95% CI [0.478, 0.776], *p* < 0.001)

In a recent investigation by Hoch et al. [8], the performance of ChatGPT in responding to questions for the otolaryngology board certification was assessed, explicitly focusing on multiple-choice and single-choice formats. They revealed that the percentage of correct responses varied based on the question format, with single-choice questions receiving a higher percentage of correct answers than did multiple-choice questions (63% vs. 34%) [8]. Furthermore, the accuracy of ChatGPT’s responses showed variation across different topics [8]. For instance, 72% of questions related to allergology were answered correctly, whereas questions about legal aspects of otolaryngology yielded a higher rate of incorrect answers (71%) [8]. Similarly, Jung et al. [10] evaluated ChatGPT’s performance on questions from the German state examination in medicine and found heterogeneity in performance across different domains, findings likely influenced by question complexity and available training data.

Our study’s findings support this observed pattern, showing variable levels of agreement on particular subtopics related to PJI of the hip and knee. Among these subtopics, questions about the prevention of PJI of the hip and knee which can be considered less complex garnered the highest mean Likert scores (4.53 ± 0.53), indicating greater reliability and usefulness. Conversely, responses as to PJI treatment and outcomes exhibited the lowest mean Likert scores (3.54 ± 0.95 and 3.61 ± 0.68, respectively). These findings align with data from Valentini et al. [18], who assessed the quality of ChatGPT’s responses to sarcoma-related questions. They revealed that ChatGPT’s performance was notably poorer in treatment-related questions, with 55% of responses classified as poor or very poor, compared to general questions (85% of responses were good or very good) and definitions (60% of responses were good or very good) [18]. Supporting the idea of the varying performance of ChatGPT based on the particular topic and the question’s complexity, Lum [16] recently demonstrated that ChatGPT’s ability to provide accurate answers to Orthopedic In-Training Examination questions declined with increasing question taxonomy and complexity, supporting our findings and the idea that ChatGPT’s performance is influenced by question complexity. Given the observed variability in the quality of AI-generated responses by ChatGPT across different subject areas [8,9,10,16], our study adds to the existing body of literature by emphasizing the importance of cautious response interpretation [14,20]. Although previous research has reported promising results for ChatGPT [9,11,16], it is crucial to avoid the assumption that AI tools that are beneficial in one subspecialty will necessarily be helpful in others [21].

A prior study by Leithner et al. [22], conducted before the ChatGPT era, examined the quality of information on osteosarcoma across various sources, including the English version of Wikipedia and the patient and health-professional versions of the National Cancer Institute’s (NCI) website. Their analysis revealed that Wikipedia was preferred due to its user-friendly interface and accessibility of patient-related content [22]. Although our experts generally perceived the free-text responses provided by ChatGPT as being suitable for patients, the findings of Leithner et al. [22] potentially emphasize the need to consider several perspectives when assessing the suitability of ChatGPT’s responses for patients and acknowledge the value of alternative sources other than ChatGPT. As no direct comparison between ChatGPT and Wikipedia has yet been conducted, an interesting project for future studies would be to examine if ChatGPT can outperform “traditional” online resources in terms of patient suitability.

This study has several limitations. First, the assessment and analysis were limited to a subset of the Hip & Knee part of the 2018 ICM consisting of 27 of its 155 questions (17.42%). As a result, the findings may not provide a comprehensive representation of ChatGPT’s performance on this specific topic. Furthermore, the study’s scope was restricted by the involvement of only three raters tasked with assessing the provided responses. The limited number of raters may have had an impact on the study’s generalizability and reliability. Moreover, it is crucial to recognize that the evaluation process relied on a subjective assessment, as the AI-generated answers were compared against the official recommendations outlined in the 2018 ICM. While efforts were made to evaluate aspects including logic and reasoning, certain subjective aspects, such as patient suitability, may introduce inherent subjectivity, particularly from a physician’s perspective. Additionally, it is crucial to note that ChatGPT is an evolving AI network, continuously learning and improving over time. As a result, if this study were to be reproduced in the future using the same methodology, its results would certainly be different. However, we attempted to minimize the impact by limiting the data collection and response-generation period to a single day to mitigate this potential bias. Therefore, it is important to consider the current study’s drawbacks when interpreting the results and to acknowledge the need for further research and refinement in evaluating ChatGPT’s performance on a wider range of questions within the field of PJIs of the hip and knee. From a formal perspective, however, our findings offer valuable insights into the quality of ChatGPT’s free-text responses to complex orthopedic questions, and this study was conducted using a solid methodology.

## 5. Conclusions

When confronted with complex questions about PJI of the hip and knee, orthopedic surgeons consider ChatGPT a valuable and comprehensive information resource for patients rather than for orthopedic surgeons. However, given ChatGPT’s early developmental stage, the authors believe there is a potential risk that it will provide free-text responses with factual errors and misleading content, particularly in specific subtopics and with increased question complexity. It is crucial to emphasize the importance of prioritizing regular updates, exercising caution when interpreting health-related data, and consulting additional sources to confirm the veracity and accuracy of the provided data.

## Figures and Tables

**Table 1:** Included questions (Q1–27) of the Hip & Knee 2018 ICM on periprosthetic joint infections (available from https://icmphilly.com/hip-knee/, accessed on 20 May 2023).

Q	Full-Text Question (Hip & Knee 2018 ICM)
Q1	What nutritional markers are the most sensitive and specific for surgical site infections and periprosthetic infections (SSIs/PJIs)? Does improvement in nutritional status reduce the risk of SSI/PJI?
Q2	What preoperative screening for infections should be performed in patients undergoing revision hip or knee arthroplasty because of presumed aseptic failure?
Q3	Should patients undergoing outpatient total joint arthroplasty (TJA) receive additional postoperative prophylactic antibiotics?
Q4	Is there a role for the use of antibiotic-impregnated cement in primary total joint arthroplasty (TJA)?
Q5	Is there a concern for contamination of the surgical field by particles, such as cement, that may escape the wound intraoperatively by coming into contact with the ceiling light or facial masks and fall back into the wound?
Q6	Does the surgical approach (parapatellar vs. subvastus) during primary total knee arthroplasty (TKA) affect the incidence of subsequent surgical site infections/periprosthetic joint infections (SSIs/PJIs)?
Q7	Can implant factors (i.e., type of bearing) influence the thresholds for serum and synovial markers in acute and chronic periprosthetic joint infections (PJIs)?
Q8	Should patients with cellulitis following total joint arthroplasty be treated with antibiotic therapy?
Q9	What clinical findings (e.g., fever, erythema, reduced range of motion) are most sensitive and specific for the diagnosis of periprosthetic joint infections (PJIs)?
Q10	Do patients with adverse local tissue reactions (ALTRs) have a higher incidence of periprosthetic joint infections (PJIs)?
Q11	Does the presence of both an erythrocyte sedimentation rate (ESR) and C-reactive protein (CRP) below the periprosthetic joint infection (PJI) thresholds rule out the diagnosis of a PJI?
Q12	Are there significant differences in the yield of culture between preoperative aspiration and intraoperative culture samples? If so, which result should be utilized?
Q13	What metrics should be considered to determine the timing of reimplantation after two-stage exchange arthroplasty of the infected hip or knee?
Q14	Is there a difference in the treatment outcome for periprosthetic joint infections (PJIs) caused by a single organism and a polymicrobial PJI?
Q15	Should patients with periprosthetic joint infections (PJIs) caused by a fungus undergo the typical two week antimicrobial holiday prior to reimplantation?
Q16	Should early postoperative infection and acute hematogenous infection be treated and managed differently?
Q17	Is debridement, antibiotics and implant retention (DAIR) an emergency procedure for patients with acute periprosthetic joint infection (PJI) or should patient optimization be implemented prior to surgery to enhance the success of this procedure?
Q18	What are the indications and contraindications for a one-stage exchange arthroplasty for the treatment of chronic periprosthetic joint infections (PJIs)?
Q19	Which antibiotic(s) should be added to a cement spacer in patients with periprosthetic joint infections (PJIs) caused by multiresistant organisms?
Q20	What is the optimal timing for reimplantation of a two-stage exchange arthroplasty of the hip and knee?
Q21	Do all metallic implants need to be removed to eradicate periprosthetic joint infections (PJIs)? Does this apply to other metal hardware present (e.g., hook plates, cables) as well?
Q22	Does the use of cemented or cementless components at the time of reimplantation affect the success of treating chronic periprosthetic joint infections (PJIs)? If yes, what is the optimal antibiotic(s), dosage and cement to maximize antibiotic delivery and mechanical properties of the cement?
Q23	What are surgical alternatives to hip disarticulation in patients with persistent joint infections?
Q24	When should rifampin be added to the regimen of antibiotics for management of patients with periprosthetic joint infections (PJIs) undergoing surgical treatment?
Q25	(A) What is the optimal length of administration for antibiotic treatment following resection arthroplasty? (B) What is the optimal mode of administration for antibiotic treatment following resection arthroplasty?
Q26	Which patients should be considered for administration of long-term suppressive oral antibiotic instead of surgical treatment in patients with chronic periprosthetic joint infections (PJIs)?
Q27	Is there a benefit for the engagement of a multidisciplinary team for the management of patients with periprosthetic joint infections (PJIs)?

**Table 2:** The reliability and usefulness of each ChatGPT response were rated using a 5-point Likert scale—as previously described in [18] and extended—on various aspects related to the provided answer to the respective question (Q1–Q27).

Aspects (Abbreviation)	Likert Scale	
Is the provided information complete? (Completeness)	5	Strongly agree
	4	Agree
	3	Neutral
	2	Disagree
	1	Strongly disagree
Is the provided answer misleading? (Misleading) *	1	Strongly agree
	2	Agree
	3	Neutral
	4	Disagree
	5	Strongly disagree
Are there relevant factual errors in the provided information? (Errors) *	1	Strongly agree
	2	Agree
	3	Neutral
	4	Disagree
	5	Strongly disagree
Is the provided information up to date? (Up-to-dateness)	5	Strongly agree
	4	Agree
	3	Neutral
	2	Disagree
	1	Strongly disagree
Is the provided answer a good source of information for patients? (Patients)	5	Strongly agree
	4	Agree
	3	Neutral
	2	Disagree
	1	Strongly disagree
Is the provided answer a good source of information for orthopedic surgeons? (Surgeons)	5	Strongly agree
	4	Agree
	3	Neutral
	2	Disagree
	1	Strongly disagree

* Reverse-transformed aspects for statistical analysis. Three independent investigators (PS, GH, and AD) evaluated each of the 27 included questions, which were obtained from the Hip & Knee subsection of the 2018 ICM. The evaluation employed a Likert-scale-type assessment ranging from 1 to 5, with corresponding descriptions encompassing a range from “Strongly agree” to “Strongly disagree”.

**Table 3:** Mean Likert scores and agreement of inter-rater reliability scores for all analyzed questions (Q1–27) based on the aspects evaluated by all three raters.

Aspects	Mean ± SD	Fleiss’ Kappa *	95% CI (Lower, Upper)	*p*
Completeness	3.80 ± 0.63	0.848	0.699, 0.996	** <0.001 **
Misleading	4.04 ± 0.67	0.743	0.601, 0.886	** <0.001 **
Errors	4.14 ± 0.58	0.880	0.724, 1.035	** <0.001 **
Up-to-dateness	3.90 ± 0.45	0.584	0.434, 0.734	** <0.001 **
Patients	3.69 ± 0.64	0.627	0.478, 0.776	** <0.001 **
Surgeons	3.63 ± 0.95	0.505	0.383, 0.628	** <0.001 **

SD, standard deviation. * <0.00 indicates poor agreement, 0.00 to 0.20 signifies slight agreement, 0.21 to 0.40 suggests fair agreement, and 0.41 to 0.60 reflects moderate agreement. Substantial agreement is denoted by a kappa value of 0.61 to 0.80, while an almost perfect agreement is indicated by kappa values ranging from 0.81 to 1.00. A *p* < 0.05 is considered statistically significant and presented in bold.

**Table 4:** Mean ± SD for survey items using a 5-point Likert scale (1—strongly disagree, 5—strongly agree; reversed for “Misleading” and “Errors”) and inter-rater reliability for each question (Q1–27) as to all three raters.

Question (Q)	Mean ± SD	Fleiss’ Kappa *	95% CI (Lower, Upper)	*p*
Q1	4.44 ± 0.51	0.775	0.313, 1.273	** 0.001 **
Q2	4.61 ± 0.61	0.182	−0.212, 0.576	0.366
Q3	4.22 ± 0.55	0.532	0.139, 0.926	** 0.008 **
Q4	4.50 ± 0.51	0.556	0.094, 1.018	** 0.018 **
Q5	5.00 ± 0.00	1.000	-	-
Q6	4.22 ± 0.43	0.357	−0.105, 0.819	0.130
Q7	4.83 ± 0.38	1.000	0.583, 1.462	** <0.001 **
Q8	4.44 ± 0.51	0.775	0.313, 1.237	** 0.001 **
Q9	4.11 ± 0.76	0.654	0.320, 0.987	** <0.001 **
Q10	2.94 ± 0.64	0.393	0.051, 0.736	** 0.024 **
Q11	4.11 ± 0.32	−0.125	−0.587, 0.337	0.596
Q12	4.11 ± 0.32	0.438	−0.024, 0.899	0.063
Q13	3.11 ± 0.32	−0.125	−0.587, 0.337	0.596
Q14	3.89 ± 0.32	0.483	−0.024, 0.899	0.063
Q15	4.28 ± 0.46	0.446	−0.016, 0.908	0.058
Q16	1.94 ± 0.42	0.234	−0.134, 0.602	0.212
Q17	3.56 ± 0.51	0.550	0.088, 1.012	** 0.020 **
Q18	4.06 ± 0.24	−0.059	−0.521, 0.403	0.803
Q19	3.72 ± 0.58	0.349	−0.048, 0.747	0.085
Q20	3.56 ± 0.51	0.775	0.313, 1.237	** 0.001 **
Q21	3.39 ± 0.70	0.811	0.446, 1.175	** <0.001 **
Q22	3.17 ± 0.71	0.273	−0.071, 0.616	0.120
Q23	3.17 ± 0.71	1.000	0.656, 1.344	** <0.001 **
Q24	4.94 ± 0.24	−0.059	−0.521, 0.403	0.803
Q25	3.33 ± 0.49	0.500	0.038, 0.962	** 0.034 **
Q26	3.11 ± 0.90	0.500	0.190, 0.810	** 0.002 **
Q27	3.61 ± 0.61	0.299	−0.095, 0.693	0.137

SD, standard deviation; Q1–27, Questions 1–27 of the included questions (Table 1); 95% CI, 95% confidence interval. * <0.00 indicates poor agreement, 0.00 to 0.20 signifies slight agreement, 0.21 to 0.40 suggests fair agreement, and 0.41 to 0.60 reflects moderate agreement. Substantial agreement is denoted by a Fleiss’ kappa of 0.61 to 0.80, while an almost perfect agreement is indicated by Fleiss’ kappa values ranging from 0.81 to 1.00. A *p* < 0.05 is considered statistically significant and presented in bold.

**Table 5:** Mean Likert scores and levels of inter-rater reliability among the three raters, including all evaluated aspects based on the six subtopics from the Hip & Knee 2018 ICM.

Subtopic	Mean ± SD	Fleiss’ Kappa *	95% CI (Lower, Upper)	*p*
Prevention (Q1–8)	4.53 ± 0.53	0.685	0.528, 0.842	**<0.001**
Diagnosis (Q9–13)	3.68 ± 0.73	0.640	0.492, 0.788	**<0.001**
Pathogen Factors (Q14)	3.89 ± 0.32	0.438	−0.024, 0.899	0.063
Fungal (Q15)	4.28 ± 0.46	0.446	−0.016, 0.908	0.058
Treatment (Q16–26)	3.54 ± 0.95	0.704	0.616, 0.792	**<0.001**
Outcomes (Q27)	3.61 ± 0.68	0.299	−0.095, 0.693	0.137

SD, standard deviation; * <0.00 indicates poor agreement, 0.00 to 0.20 signifies slight agreement, 0.21 to 0.40 suggests fair agreement, and 0.41 to 0.60 reflects moderate agreement. Substantial agreement is denoted by a Fleiss’ kappa of 0.61 to 0.80, while an almost perfect agreement is indicated by Fleiss’ kappa values ranging from 0.81 to 1.00. A *p* < 0.05 is considered statistically significant and presented in bold.

## Data Availability

The data that support the findings of this study are available upon reasonable request from the corresponding author.

## Supplementary Materials

The following supporting information can be downloaded at: https://www.mdpi.com/article/10.3390/jcm12206655/s1, Table S1: Mean ± SD for survey items using a 5-point Likert scale (1-strongly disagree, 5-strongly agree; reversed for “Misleading” and “Errors”) scores and inter-rater reliability for each question (Q1–27) of all three raters based on evaluated aspects.
