# Supplementary material for: Are ChatGPT’s Free-Text Responses on Periprosthetic Joint Infections of the Hip and Knee Reliable and Useful?
Source: J Clin Med. 2023 Oct 20;12(20):6655. doi: 10.3390/jcm12206655 (PMC10607052; doi:10.3390/jcm12206655)
Supplement: Supplementary file 1 [file jcm-12-06655-s001.zip › jcm-2607461-supplementary.pdf]

Table S1. Mean ± SD for survey items using a 5-point Likert scale (1-strongly disagree, 5-strongly agree; reversed for "Misleading" and "Errors") scores and inter-rater reliability for each question (Q1-27) of all three raters based on evaluated aspects.

Question (Q)	Aspects	Likert scale Rater #1	Rater #2	Rater #3	Mean ± SD	Fleiss' kappa* (95% CI [lower, upper])	*p*
Q1					4.44 ± 0.51	0.775 [0.313, 1.273]	**0.001**
	Completeness	4	4	4			
	Misleading	4	5	5			
	Errors	5	5	5			
	Up-to-dateness	4	4	4			
	Patients	5	5	5			
	Surgeons	4	4	4			
Q2					4.61 ± 0.61	0.182 [-0.212, 0.576]	0.366
	Completeness	4	5	4			
	Misleading	5	5	5			
	Errors	5	5	5			
	Up-to-dateness	4	5	4			
	Patients	3	5	4			
	Surgeons	5	5	5			
Q3	Completeness	4	4	4	4.22 ± 0.55	0.532 [0.139, 0.926]	**0.008**
	Misleading	5	4	5			
	Errors	5	5	5			
	Up-to-dateness	4	4	4			
	Patients	3	4	4			
	Surgeons	4	4	4			
Q4	Completeness	4	5	5	4.50 ± 0.51	0.556 [0.094, 1.018]	**0.018**
	Misleading	5	5	5			
	Errors	5	5	5			
	Up-to-dateness	4	4	4			
	Patients	4	5	4			
	Surgeons	4	4	4			
Q5	Completeness	5	5	5	5.00 ± 0.00	1.000	-
	Misleading	5	5	5			
	Errors	5	5	5			
	Up-to-dateness	5	5	5			
	Patients	5	5	5			
	Surgeons	5	5	5			
Q6	Completeness	4	4	4	4.22 ± 0.43	0.357 [-0.105, 0.819]	0.130
	Misleading	5	4	5			
	Errors	4	4	4			
	Up-to-dateness	4	4	4			
	Patients	4	4	4			
	Surgeons	5	4	5			
Q7	Completeness	5	5	5	4.83 ± 0.38	1.000 [0.583, 1.462]	**<0.001**
	Misleading	5	5	5			
	Errors	5	5	5			
	Up-to-dateness	5	5	5			
	Patients	4	4	4			

	Surgeons	5	5	5			
Q8	Completeness	4	4	4	4.44 ± 0.51	0.775 [0.313, 1.237]	**0.001**
	Misleading	5	5	5			
	Errors	5	5	5			
	Up-to-dateness	4	4	4			
	Patients	4	4	4			
	Surgeons	5	4	5			
Q9	Completeness	3	3	4	4.11 ± 0.76	0.654 [0.320, 0.987]	**<0.001**
	Misleading	5	5	5			
	Errors	5	5	5			
	Up-to-dateness	4	4	4			
	Patients	4	4	4			
	Surgeons	3	3	4			
Q10	Completeness	3	3	3	2.94 ± 0.64	0.393 [0.051, 0.736]	**0.024**
	Misleading	2	2	2			
	Errors	2	3	3			
	Up-to-dateness	4	3	3			
	Patients	4	4	3			
	Surgeons	3	3	3			
Q11	Completeness	4	4	4	4.11 ± 0.32	-0.125 [-0.587, 0.337]	0.596
	Misleading	4	4	4			
	Errors	4	4	4			
	Up to date	5	4	4			
	Patients	4	4	4			
	Surgeons	5	4	4			
Q12	Completeness	4	4	4	4.11 ± 0.32	0.438 [-0.024, 0.899]	0.063
	Misleading	4	4	4			
	Errors	4	4	4			
	Up-to-dateness	4	4	4			
	Patients	4	4	4			
	Surgeons	5	4	5			
Q13	Completeness	3	3	3	3.11 ± 0.32	-0.125 [-0.587, 0.337]	0.596
	Misleading	3	3	4			
	Errors	3	3	3			
	Up-to-dateness	3	3	3			
	Patients	3	3	3			
	Surgeons	4	3	3			
Q14	Completeness	4	4	4	3.89 ± 0.32	0.483 [-0.024, 0.899]	0.063
	Misleading	4	4	4			
	Errors	4	4	4			
	Up-to-dateness	4	4	4			
	Patients	3	4	3			
	Surgeons	4	4	4			
Q15	Completeness	5	5	5	4.28 ± 0.46	0.446 [-0.016, 0.908]	0.058
	Misleading	5	4	4			
	Errors	4	4	4			
	Up-to-dateness	4	4	4			
	Patients	4	4	4			
	Surgeons	5	4	4			
Q16	Completeness	2	2	2	1.94 ± 0.42	0.234 [-0.134, 0.602]	0.212
	Misleading	2	2	2			

	Errors	2	2	3			
	Up-to-dateness	2	2	2			
	Patients	2	2	2			
	Surgeons	2	1	1			
Q17	Completeness	3	3	3	3.56 ± 0.51	0.550 [0.088, 1.012]	**0.020**
	Misleading	4	4	3			
	Errors	4	4	4			
	Up-to-dateness	4	4	3			
	Patients	4	4	4			
	Surgeons	3	3	3			
Q18	Completeness	4	4	4	4.06 ± 0.24	-0.059 [-0.521, 0.403]	0.803
	Misleading	4	4	4			
	Errors	4	4	4			
	Up-to-dateness	4	4	4			
	Patients	4	4	4			
	Surgeons	5	4	4			
Q19	Completeness	3	3	4	3.72 ± 0.58	0.349 [-0.048, 0.747]	0.085
	Misleading	4	4	4			
	Errors	4	4	4			
	Up-to-dateness	5	4	4			
	Patients	3	3	3			
	Surgeons	4	3	4			
Q20	Completeness	4	4	4	3.56 ± 0.51	0.775 [0.313, 1.237]	**0.001**
	Misleading	3	3	3			
	Errors	3	3	3			
	Up-to-dateness	4	4	4			
	Patients	4	4	4			
	Surgeons	3	3	4			
Q21	Completeness	3	3	3	3.39 ± 0.70	0.811 [0.446, 1.175]	<0.001
	Misleading	4	4	4			
	Errors	4	4	4			
	Up-to-dateness	4	4	4			
	Patients	3	3	3			
	Surgeons	2	2	3			
Q22	Completeness	3	3	3	3.17 ± 0.71	0.273 [-0.071, 0.616]	0.120
	Misleading	4	3	4			
	Errors	4	4	4			
	Up-to-dateness	3	3	4			
	Patients	2	3	3			
	Surgeons	2	2	3			
Q23	Completeness	3	3	3	3.17 ± 0.71	1.000 [0.656, 1.344]	<0.001
	Misleading	3	3	3			
	Errors	4	4	4			
	Up-to-dateness	4	4	4			
	Patients	3	3	3			
	Surgeons	2	2	2			
Q24	Completeness	5	5	5	4.94 ± 0.24	-0.059 [-0.521, 0.403]	0.803
	Misleading	5	5	5			
	Errors	5	5	5			
	Up-to-dateness	5	5	5			
	Patients	4	5	5			

	Surgeons	5	5	5			
Q25	Completeness	4	4	4	3.33 ± 0.49	0.500 [0.038, 0.962]	0.034
	Misleading	3	3	3			
	Errors	4	4	3			
	Up-to-dateness	3	3	4			
	Patients	3	3	3			
	Surgeons	3	3	3			
Q26	Completeness	3	3	3	3.11 ± 0.90	0.500 [0.190, 0.810]	0.002
	Misleading	4	4	4			
	Errors	4	4	4			
	Up-to-dateness	3	3	4			
	Patients	2	3	3			
	Surgeons	1	2	2			
Q27	Completeness	4	4	4	3.61 ± 0.61	0.299 [-0.095, 0.693]	0.137
	Misleading	4	4	4			
	Errors	4	4	4			
	Up-to-dateness	3	3	4			
	Patients	3	4	4			
	Surgeons	2	3	3			

SD, standard deviation; Q1-27, Question 1-27 of the included questions (Table 1); 95% CI, 95% confidence interval. *<0.00 indicates poor agreement, 0.00 to 0.20 signifies slight agreement, 0.21 to 0.40 suggests fair agreement, and 0.41 to 0.60 reflects moderate agreement. Substantial agreement is denoted by a Fleiss' kappa of 0.61 to 0.80, while an almost perfect agreement is indicated by a Fleiss' kappa ranging from 0.81 to 1.00. A p<0.05 is considered statistically significant and presented in bold.
